# Cadherin 17 and digestive cancers: from diagnostic to therapeutic opportunities

**DOI:** 10.1186/s13046-026-03693-8

**Published:** 2026-04-02

**Authors:** Benjamin Fernandez, Léa Lopez, Thibaut Matis, Sandrine Dabernat, Samuel Amintas

**Affiliations:** 1 Digestive Surgery Department, CHU Bordeaux, Pessac, F-33600 France; 2 Cancer Genetics Unit, Institut Bergonié, Bordeaux, F-33000 France; 3 Biochemistry Laboratory, CHU Bordeaux, Bordeaux, F-33000 France; 4 Tumor Biology and Tumor Bank Laboratory, CHU Bordeaux, Pessac, F-33600 France; 5 BRIC (BoRdeaux Institute of onCology), UMR1312, INSERM, University of Bordeaux, Bordeaux, F- 33000 France

**Keywords:** Cadherin 17, CDH17, LI-Cadherin, Digestive cancers, Biomarker, Therapeutic target

## Abstract

Cadherin-17 (CDH17, LI-cadherin) is a non-classical cadherin with restricted expression in normal gastrointestinal epithelium, emerging as a multifunctional molecule in cancer. CDH17 regulates cell–cell adhesion, modulates key signaling pathways, and interacts with the tumor microenvironment, collectively influencing proliferation, invasion, metastasis, therapeutic resistance, and immune evasion. Its expression is highly context-dependent and dynamically regulated, with both upregulation and loss linked to distinct features of tumor aggressiveness and differentiation. CDH17 also interfaces with pathways governing stemness and cellular plasticity, suggesting a role in modulating therapeutic response and resistance mechanisms.

Beyond its biological functions, CDH17 has been investigated as a diagnostic marker, with tissue-based detection, circulating biomarkers, and radiolabeled imaging probes exploiting its tumor-restricted expression and membrane localization, offering opportunities for noninvasive tumor detection, staging, and monitoring. CDH17 also holds potential prognostic significance, although its clinical relevance varies according to molecular context and tumor differentiation status, emphasizing the need for integrative biomarker assessment. Finally, the tumor-specific expression of CDH17 has inspired multiple therapeutic strategies, including cellular immunotherapies, antibody–drug conjugates, immunotoxins, radiolabeled agents, and engineered delivery platforms, all designed to selectively target CDH17-expressing cells while minimizing off-target toxicity. Such strategies highlight the translational potential of CDH17 as both a therapeutic target and a platform for precision oncology.

In this review, we summarize the molecular mechanisms, biological functions, diagnostic and prognostic relevance, and therapeutic applications of CDH17. By integrating current findings and addressing existing challenges, we aim to provide a comprehensive overview of its multifaceted roles and to emphasize emerging strategies to harness this molecule for clinical applications in cancer.

## Background

Cadherins are a superfamily of calcium-dependent transmembrane glycoproteins that regulate cell–cell adhesion and tissue architecture [1]. Beyond their structural role, they actively participate in signaling pathways controlling proliferation, differentiation, and cellular plasticity. Dysregulation of cadherin networks is a hallmark of carcinogenesis, notably through epithelial–mesenchymal transition, where loss of E-cadherin and gain of N-cadherin promote invasive phenotypes [1, 2]. However, the biological functions of individual cadherins are highly context-dependent and extend beyond classical adhesion dynamics.

Among the cadherin superfamily, Cadherin-17 (CDH17), also known as liver–intestinal cadherin (LI-cadherin), represents a structurally and functionally atypical member. In contrast to classical cadherins, CDH17 lacks the canonical cytoplasmic catenin-binding domain and therefore does not directly connect to the actin cytoskeleton through β-catenin. This structural particularity suggests distinct adhesive complexes and signaling properties compared with conventional cadherins, positioning CDH17 as a non-classical cadherin with specialized functions.

Physiologically, CDH17 expression is largely restricted to intestinal epithelial cells and tightly regulated by CDX2, a master transcription factor governing intestinal lineage specification. This lineage-associated regulation distinguishes CDH17 from broadly expressed cadherins frequently involved in epithelial-mesenchymal transition. In tumor contexts, CDH17 expression often parallels intestinal differentiation programs, linking it more closely to lineage identity than to generic EMT switching.

In digestive tumor contexts, CDH17 has been implicated in tumor-associated signaling networks, including Wnt/β-catenin and integrin-dependent pathways, and has been associated with proliferation, migration, stemness, and resistance to therapy. Its expression appears dynamically regulated during tumor progression, being frequently preserved in well-differentiated tumors and altered in advanced or dedifferentiated contexts.

Importantly, CDH17 combines several features of translational interest: restricted physiological distribution, membranous accessibility, and functional involvement in oncogenic signaling. These characteristics have supported its evaluation as a diagnostic marker, a prognostic indicator, and, more recently, as a therapeutic target. Indeed, its extracellular accessibility has enabled the development of multiple CDH17-directed strategies, including monoclonal antibodies, antibody–drug conjugates, CAR-T and CAR-NK approaches, and radiolabeled tracers with theranostic potential.

Together, these features position CDH17 as a lineage-associated and functionally active surface molecule with increasing biological and translational significance in digestive malignancies. In this context, the present review synthesizes current knowledge on CDH17, from its molecular and biological properties to its diagnostic, prognostic, and emerging therapeutic implications.

## Cadherin 17 overview

### Genetic and structural aspects

**Fig. 1:**
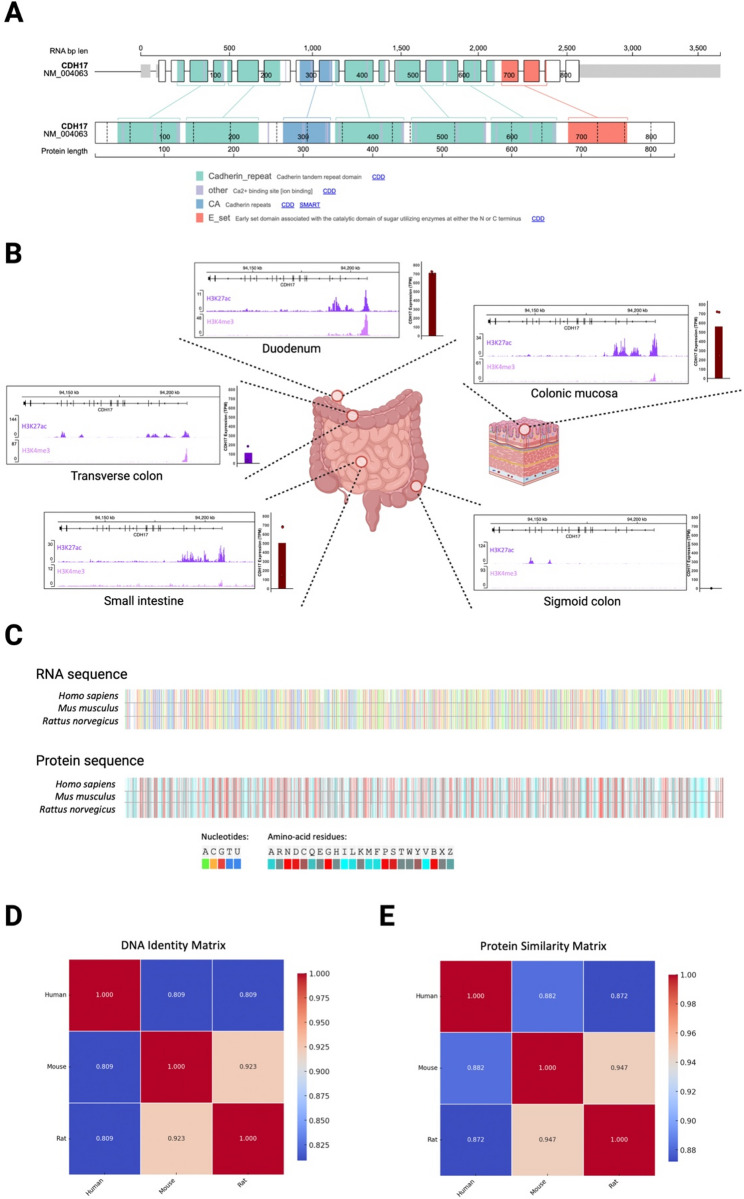
Genomic, transcriptional, and translational features of CDH17. **A** Schematic representation of the human *CDH17* canonical isoform (NM_004063.4). It consists of 3670 base pairs and encodes a protein of 833 amino acids. **B** Expression profile of *CDH17* across the gastrointestinal tract based on publicly available epigenomic (ChIP-Seq, ENCODE database) and transcriptomic (Total RNA-seq, ENCODE, Gtex, Human Protein Atlas datasets) (**C**) RNA and proteins *sequence* alignment of *CDH17* among human, mouse, and rat, showing high interspecies conservation (**D**) *CDH17* DNA identity matrix among human, mouse, and rat. **E** CDH17 protein similarity matrix among human, mouse, and rat. Fig. 1B was created with BioRender.com

The *CDH17* gene, located on human chromosome 8q22.1, contains 22 exons and 14 alternative transcripts. The canonical isoform (NM_004063.4) comprises 3670 base pairs and encodes 833 amino acids (Fig. 1A). In humans, *CDH17* expression is mainly regulated by the transcription factor CDX2. CDX2 is a homeobox transcription factor that binds DNA and is primarily expressed in digestive tissues. It plays a key role in intestinal maintenance and development. CDX2 regulates *CDH17* transcription by binding to its 5’-flanking region [3]. Consequently, *CDH17* expression is limited to gastrointestinal (GI) tract tissues. The transcription factor HNF1α has also been identified as a regulator of *CDH17* expression by binding to the promoter-proximal region, particularly in hepatocellular carcinoma [4]. Fig. 1B was obtained from epigenetics (ChIP-Seq) and transcriptomics (Total RNA-seq) public databases, illustrating the expression pattern of the *CDH17* gene across the GI tract. Interestingly, according to those genetic data, *CDH17* expression is higher in the duodenum, small intestine, and proximal colon, while it is decreased in the transverse colon and virtually absent in the sigmoid colon, suggesting a spatial expression gradient along the intestinal tract (Fig. 1B). However, additional data, particularly tissue-level protein expression, would be required to substantiate this observation, given that CDH17 is generally regarded as being expressed throughout the entire large intestine.

As the human *CDH17*, the 3.6 kb transcript of rat *Cdh17* encodes a protein of approximately 92 kDa. However, on western blot analysis using a CDH17-specific antibody for both human and rodents, the molecular mass of CDH17 increases to approximately 120–130 kDa due to the presence of seven N-glycosylation sites [5]. A comparative in silico structural analysis using EMBOSS Cons software reveals a high degree of conservation among rat, mouse, and human sequences. Mouse and rat isoforms exhibit 92.3% identity and similarity (Fig. 1C). In contrast, comparisons between rodent (mouse/rat) and human isoforms show 80.9% identity and similarity (Fig. 1D). At the protein level, the results are nearly identical, with comparable percentages for both identity and similarity (Fig. 1E).

**Fig. 2:**
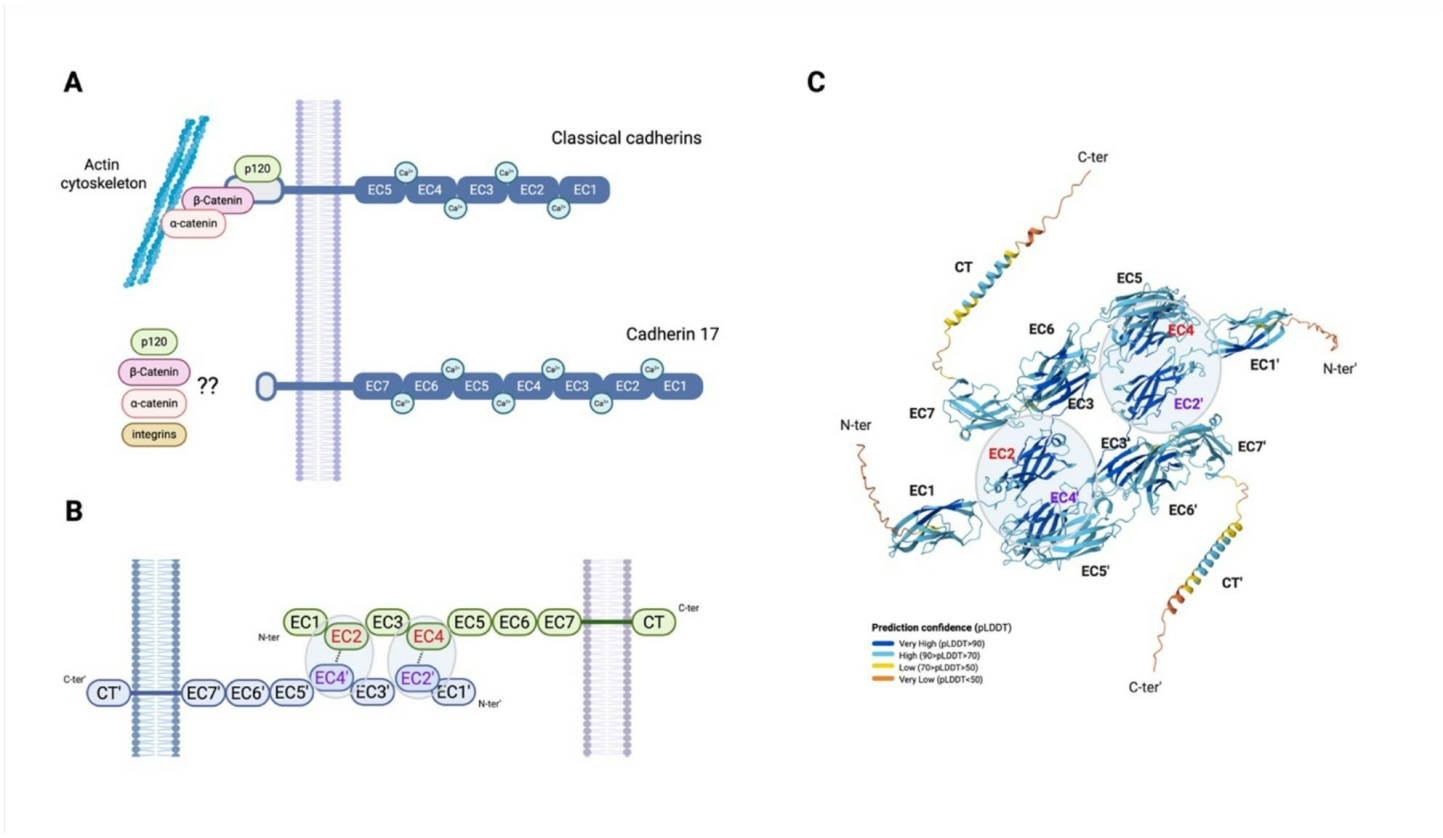
Structural and interactional features of CDH17. **A** Comparative structural organization and interaction patterns of conventional cadherins versus cadherin-17; (**B**) *CDH17* homodimerization mediated by interactions between extracellular cadherin-like domains EC2 and EC4 (indicated by dotted lines); (**C**) AlphaFold^®^-based predictive model of CDH17–CDH17 homodimerization.EC: extracellular domain; CT: cytoplasmic domain; C-ter: C-terminal domain; N-ter: N-terminal domain. Fig. 1A and B were created with BioRender.com

Despite its denomination, cadherin 17, also called liver intestinal cadherin (LI-cadherin), belongs to the cadherin superfamily, but it is distinguished from the so-called classic cadherins by its structural and functional features [6, 7]. Classical cadherins are transmembrane glycoproteins characterized by five extracellular cadherin repeats (EC domains), a transmembrane domain, and a cytoplasmic tail (Fig. 2A). The EC domains mediate homophilic interactions between cadherin molecules on adjacent cells, forming adhesive contacts crucial for cell-cell adhesion and tissue integrity. Through the formation of adherent junctions, this domain establishes connections with the actin cytoskeleton and regulates cellular processes, including tissue development [1]. If like classical cadherin, calcium is also essential for stabilization of the CDH17 extracellular domain, allowing homophilic adhesion, its extracellular domain consists of seven extracellular cadherin repeats (EC1-7) instead of five (EC1-5) (Fig. 2A) [1]. This difference is explained by a putative common origin of E-cadherin and CDH17 [8]. Indeed, Jung et al.. show that the intron-exon structure is fully conserved between CDH17 repeats 3–7 and classical cadherins 1–5. Repeats 1–4 of CDH17 also show strong genomic similarity to classical cadherin repeats 1–2. This analysis supports the idea that CDH17 arose from a five-domain ancestral cadherin via partial gene duplication. To elucidate CDH17 homodimerization mechanisms, Yui et al. studied the crystal structure of the LI-cadherin homodimer containing the first four extracellular cadherin repeats [9] Authors report that EC1-4 homodimer exhibited a unique architecture, different from that of other cadherins, and driven by the interactions between EC2 and EC4 of 2 CDH17 proteins (Fig. 2B). In addition, the phenylalanine in position 244 appeared essential for CDH17 homodimerization, and the crystal structure also revealed a non-canonical calcium ion-free link between the EC2 and EC3 domains. Those results are confirmed by the recently developed Alphafold3^®^ algorithm [10], which predicts a homophylic interaction between the 4 first four EC domains (EC 1 to 4), with a high to very high confidence (Fig. 2C). Interestingly, another study highlighted the lack of tryptophan in the EC1 domain, an amino acid shown to be essential for conventional cadherin dimerization [11]. Authors also reported the presence of unique features in CDH17 EC1-2, rendering it incompatible with the classical cadherins’ EC1 strand-swap adhesion mechanism.

Next to EC domains, conventional cadherins have a cytoplasmic tail (typically around 150 amino acids) which interacts with cytoskeletal proteins and signaling molecules, modulating intracellular signaling pathways and cellular processes [12, 13]. Stabilization interaction occurs in the juxtamembrane region of cadherins, where it interacts with p120 catenin family (like CTNND1), while the catenin-binding domain associates with β-catenin (Fig. 2A) [1]. Compared to classical cadherins, the CDH17 cytoplasmic domain is relatively short, with 20 amino acids compared to the typical 150 amino acids present for other conventional cadherins [14], lacking catenin binding sites (Fig. 2A). The interactions of this short domain with intracellular effectors have not been thoroughly investigated under physiological conditions. However, multiple mechanisms of action and interaction pathways involving CDH17 have been characterized in various models of digestive system tumors (see below). Additionally, this short cytoplasmic tail of CDH17 tends to facilitate the lateral mobility for CDH17 in the plasma membrane [15], and consequently cellular adhesion modulation [16]. Interestingly, kidney-specific cadherin 16 (CDH16) also harbors these unique extracellular and intracytoplasmic structural features [17]. In consequence, CDH16 and CDH17 form the cadherin 7D subfamily.

### Tissue expression and embryonic development

In rodents, Cdh17 has a restricted pattern of tissue expression. In rats, it is abundantly expressed in the liver, in particular on the basolateral surface of hepatocytes, and in the small intestine. In contrast, mouse Cdh17 is highly expressed in the small intestine and colon, with no detectable levels in other organs such as the liver and stomach. As mentioned before, human *CDH17* is selectively expressed only on enterocytes and goblet cells in the small and large intestine [18]. More precisely, CDH17 expression localizes in intestinal epithelial cells and the basolateral membrane of enterocytes and has been associated with lipid rafts [19]. Consequently, CDH17 and the classical E-cadherin are the predominant two cadherins in the intestinal epithelium [20]. During mouse embryonic development, Cdh17 is initially detected on embryonic day (E) 11.5. Fetal Cdh17 first appears in the intestines on E12.5 and can still be detected until E16.5 [5]. Detectable expression of Cdh17 is also found in E13.5 and E16.5 mouse livers, but not detected in postnatal. During Zebrafish embryogenesis, the cdh17 ortholog is solely expressed in the pronephric ducts and in the mesonephros during larval development and adulthood [21]. As its human ortholog, liver and intestinal tissues expressed Cdh17. Additionally, depletion of Cdh17 function using antisense morpholino oligonucleotides compromised cell cohesion during pronephric duct formation, indicating an essential function during embryogenesis [21]. Very few data are available regarding *CDH17* expression modulation during human embryonic development. However, a transcriptomic analysis of embryonic intestinal tissues revealed a continuous increase in *CDH17* expression from weeks 10 to 20, concomitant with the CDX2 expression. No expression of *CDH17* and CDX2 was observed in other embryonic tissues, except for a minimal expression in stomach tissues [22, 23].

### Physiological functions

CDH17 is a calcium-dependent transmembrane glycoprotein that mediates cell-cell adhesion in the intestinal epithelium [1]. Cadherin 17 was originally cloned from rat liver and identified as a novel calcium-dependent cell adhesion molecule, present exclusively in the intestine and liver, which led to its designation as LI-cadherin [6]. Two months earlier, Dantzig et al.. described HPT-1, an approximately 92-kilodalton membrane protein that was associated with the acquisition of peptide transport activity by transport-deficient cells [7]. This protein facilitated the oral absorption of peptide-based drugs such as beta-lactam antibiotics and angiotensin-converting enzyme inhibitors. The authors further indicated that HPT-1 retains several conserved structural features of classical cadherins, including extracellular internal repeats, characteristic short amino acid motifs, conserved cysteine residues, and a transmembrane domain, despite low overall sequence identity and the absence of the conserved cytoplasmic region. Together, these two studies constitute the foundational work underpinning subsequent investigations into the structure and function of CDH17.

Similar to classical cadherins, CDH17 can mediate homotypic interactions with neighboring cells. However, in intestinal epithelial cells, CDH17 also engages in heterotypic trans-interactions with E-cadherin, exhibiting comparable binding strength to their respective homotypic interactions [15]. Notably, CDH17 is predominantly localized in cholesterol-rich membrane domains and along the basolateral membrane of enterocytes, whereas E-cadherin is excluded from these domains and mainly concentrated at adherent junctions. These observations suggest that CDH17–E-cadherin interactions may play a role during early stages of intestinal epithelial development, before the establishment of distinct membrane specializations. The adhesive mechanism of CDH17 also differs from that of classical cadherins [15, 20]. Indeed, classical cadherins require interaction with α- and β-catenin, which are adaptor molecules for anchoring actin filaments, whereas CDH17 can retain its adhesive function without interacting with other cytoplasmic components [24, 25]. Due to the organization and orientation of different junctions between enterocytes, human CDH17 is likely intermingled with other junctional proteins, such as those in adherent junctions, desmosomes, and gap junctions, located on the same surface of enterocytes (and hepatocytes in rats) [26, 27]. Unlike classical cadherins, it ensures calcium-dependent cell–cell adhesion without relying on catenins or the actin cytoskeleton, indicating a complementary role that remains active even when classical cadherin function is reduced [28]. Similarly, CDH17 promotes cell–cell adhesion through conventional dynamic trans-interactions and stable cis-dimerization, independently of tryptophan 2 in its first extracellular domain, which sets its adhesive mechanism apart from that of classical cadherins [29]. Additionally, Wendeler et al. described CDH17 binding properties as highly sensitive to extracellular calcium concentrations. Thus, CDH17 might serve as a calcium-regulated switch for the adhesive system on basolateral membranes of the intestinal epithelium [20]. This calcium-sensing function can also potentially serve to regulate the direction and efficiency of epithelial water transport via trans-interaction with cadherins of neighboring cells [30, 31]. Additionally, its Ca²⁺-dependent E-cadherin adhesion regulation further suggests a role in osmotic regulation [15, 19]. However, while the full spectrum of physiological functions of CDH17 is still being explored, its potential implications in pathological conditions, including cancer, also warrant further investigation.

## Cadherin 17 in cancer biology

### Tumor tissue expression

In physiological conditions, CDH17 expression is largely restricted to gastrointestinal epithelial tissues. The first report of CDH17 overexpression in rat liver tumor tissue was made by Hixson et al. in 1989, prior to the formal description and naming of CDH17 [32]. This overexpression is frequently associated with tumoral CDX2 overexpression [3, 33]. Subsequently, increased CDH17 expression has been consistently observed in colorectal tumor tissues, and neoexpression has been highlighted in multiple other digestive tumors [34].

A large tissue microarray study analyzing over 18,000 tumor and non-tumor samples demonstrated that CDH17 expression is highly restricted to GI tissues under physiological conditions and markedly upregulated in a subset of epithelial tumors. Notably, CDH17 was strongly expressed in 98% of colorectal adenocarcinomas [35]. Its expression was also observed in gastric (38–57%), pancreatic (40.5%), and biliary (24.5%) adenocarcinomas. More specifically, CDH17 expression is usually diffuse and strong in colorectal adenocarcinomas, whereas it is typically focal or scattered in adenocarcinomas of the stomach, pancreas, and bile duct, and largely absent in tumors of other sites [35].

CDH17’s involvement in the tumorigenesis of gastrointestinal cancers is increasingly recognized. Its expression appears to be dynamically regulated during cancer progression: it is frequently maintained or upregulated in early-stage, well-differentiated tumors and then reduced as tumors advance, undergo epithelial–mesenchymal transition (EMT), and acquire a poorly differentiated phenotype [36–39]. Additionally, *CDH17* expression is mainly regulated by CDX2, its principal described transcription factor, consistent with its association with intestinal differentiation programs [40].

**Fig. 3:**
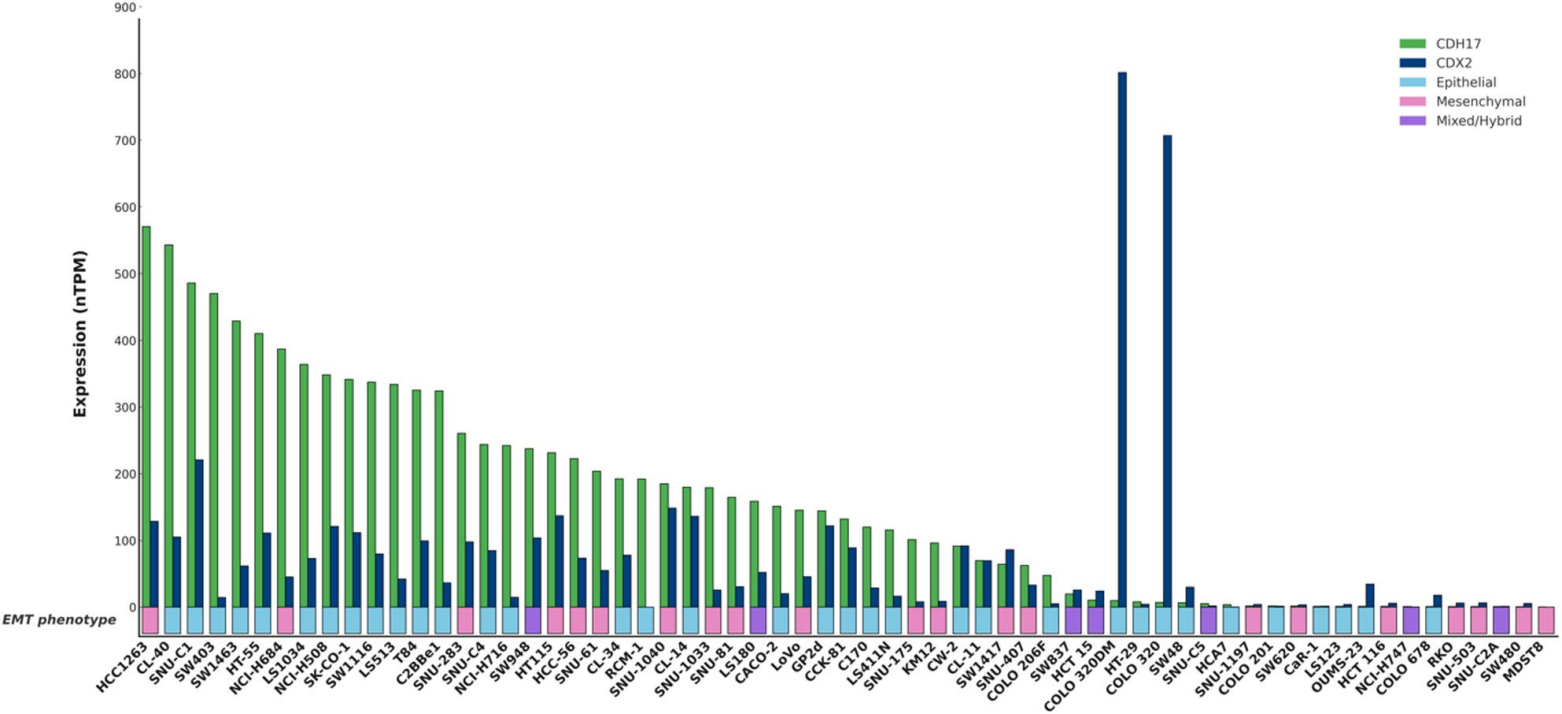
CDH17 and CDX2 expression in relation to EMT phenotypes in colorectal cancer cell lines. Normalized transcriptomic expression (nTPM) of CDH17 and CDX2 across colorectal cancer cell lines ranked by decreasing CDH17 levels. EMT phenotypes are indicated for each model (epithelial, mesenchymal, mixed/hybrid)

To further illustrate the heterogeneity of *CDH17/CDX2* expression and its association with colorectal tumor epithelial or mesenchymal phenotypes, we analyzed publicly available transcriptomic data across a panel of colorectal cancer cell lines (Human Protein Atlas, proteinatlas.org). As shown in Fig. 3, *CDH17* and *CDX2* expression levels display substantial variability across models but follow a similar pattern, with higher levels predominantly associated with an epithelial phenotype, whereas their progressive decrease parallels the emergence of hybrid and fully mesenchymal EMT states. This pattern supports the concept that loss of *CDH17* expression accompanies epithelial–mesenchymal plasticity rather than constituting a strict EMT marker. However, Fig. 3 also illustrates that *CDH17* and *CDX2* expression patterns may be dissociated in certain tumor contexts, indicating the involvement of alternative regulatory mechanisms. In line with this, CDH17 has been identified as a downstream effector of the HOXA13 transcription factor, contributing to modulation of the Wnt/β-catenin signaling pathway in gastric cancer [41]. Additionally, CDH17 gene amplifications have been described in gastric cancer, suggesting that tumor upregulation may be linked not only to transcriptional modulation but also to genetic events [42]. Together, these observations indicate that CDH17 expression is governed by a complex and dynamic transcriptional network reflecting both differentiation status and tumor plasticity.

**Fig. 4:**
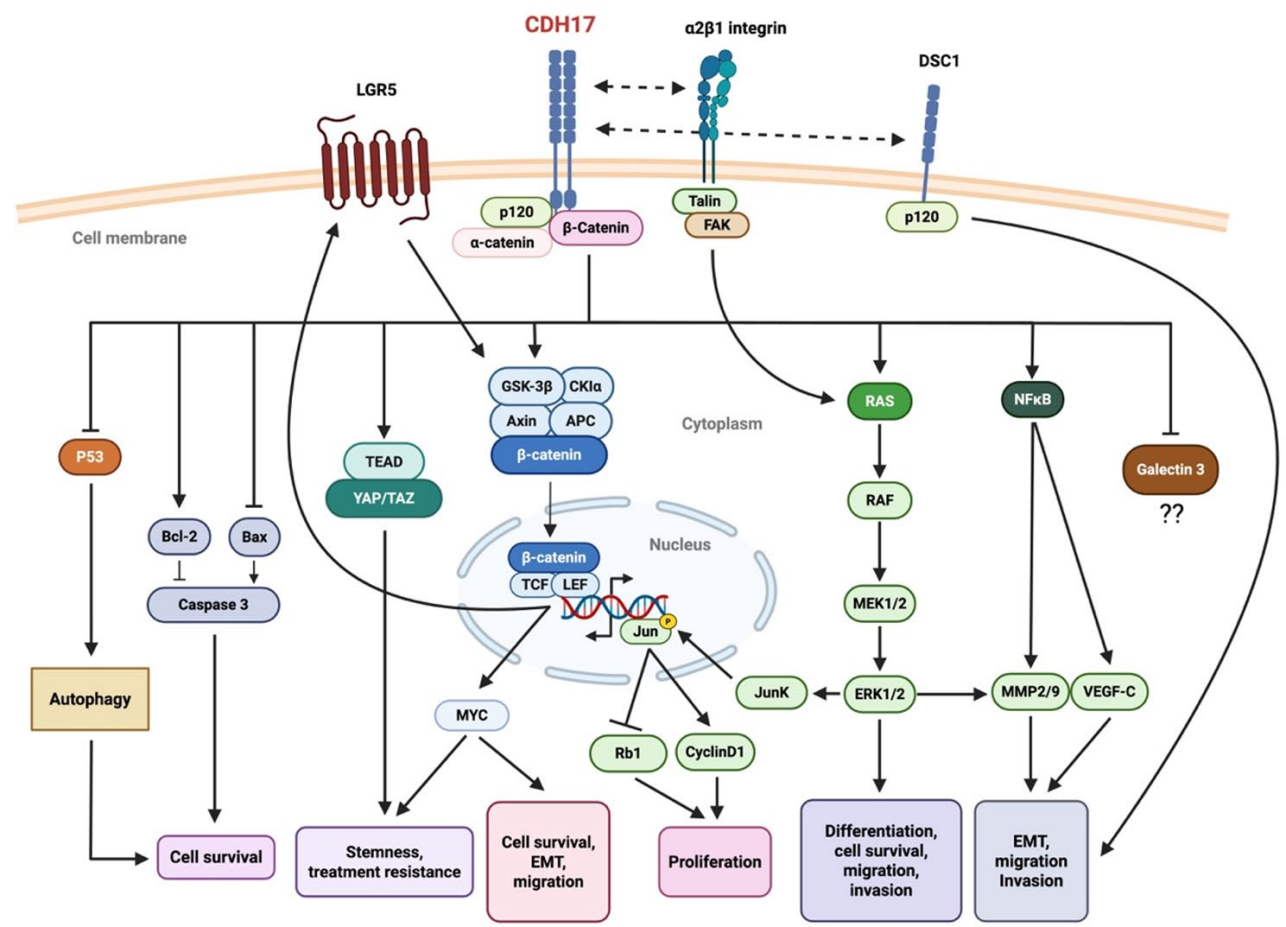
Cellular signaling networks associated with CDH17 in tumor biology. Illustration of the main signaling pathways regulated or influenced by CDH17 in a tumor context. CDH17 interacts with membrane-associated partners such as p120-catenin and α or β-catenin, modulating intracellular complexes involved in Wnt/β-catenin signaling, MAPK cascade, and PI3K/AKT pathway. CDH17 promotes nuclear translocation of β-catenin, leading to the transcriptional activation of genes involved in cell survival, migration, EMT, invasion, and proliferation (*MYC*, *CCND1*). CDH17 also affects the regulation of autophagy and apoptosis processes, leading to increased cell survival. CDH17 also contributes to stemness and treatment resistance, notably through the promotion of the expression of the stem cell marker LGR5 (in CRC) and its interaction with the YAP signaling pathway (LUAD). Additional interactions with membrane proteins (α2β1 integrin, DSC1) and signaling mediators (NFκB, galectin-3) contribute to the complexity of CDH17-associated oncogenic mechanisms. EMT: Epithelial mesenchymal transition. Figure created with BioRender.com

Beyond its individual regulation, CDH17 is consistently co-expressed with E-cadherin in normal intestinal epithelium and frequently co-expressed in tumors, with evidence that these two cadherins can interact [15, 19]. This suggests a potential synergistic or complementary role within cadherin networks. The frequent loss of E-cadherin during tumor progression may disrupt this interaction and alter CDH17 localization or signaling functions, potentially contributing to tumor invasiveness. These observations underscore the importance of considering cadherin networks rather than individual molecules when analyzing tumor biology.

Although predominantly associated with digestive malignancies, CDH17 neoexpression has also been identified in selected non-digestive tumors. In a cohort of 182 epithelial ovarian cancer (EOC) cases, including various histological types (serous, mucinous, clear cell, and endometrioid adenocarcinoma), IHC analyses revealed marked CDH17 overexpression in approximately 78% of tumor tissues, whereas its presence was nearly undetectable in normal ovarian epithelium [43]. CDX2 was frequently downregulated in these tumors and exhibited a negative correlation with CDH17 expression. Consistent with these findings, data from the Human Protein Atlas confirm elevated CDH17 expression in this subtype of ovarian cancer [44]. CDH17 expression has also been documented in mucinous epithelial ovarian tumors [45]. More recently, CDH17 expression has been reported in lung adenocarcinoma (LUAD) tissues [46, 47], in extracellular vesicles derived from LUAD [48], and in LUAD circulating tumor cells [49].

### Signaling network interactions and oncogenic implications

Among the relatively limited number of CDH17-focused mechanistic studies, a consistent picture is progressively emerging: CDH17 does not merely participate in tumor biology, but appears to sit at the intersection of adhesion dynamics and intracellular signaling cascades, shaping tumor progression. Fig. 4 summarizes the main cellular signaling networks associated with CDH17 in tumor biology.

A recurring mechanistic theme across digestive tumor models is the functional connection between CDH17 and the Wnt/β-catenin pathway. In their recent systematic review and meta-analysis, Shrestha et al. conclude that, rather than acting as a passive adhesion molecule, CDH17 acts as an upstream regulator of canonical Wnt/β-catenin signaling across multiple digestive cancers, including colorectal, gastric, and liver tumors, and emphasizing that CDH17 inhibition consistently reduces β-catenin activity, downstream proliferative signaling, and tumor growth in preclinical models [50]. More specifically in gastric cancer (GC), CDH17 silencing led to reduced β-catenin expression and decreased GSK-3β phosphorylation, while restoration of CDH17 rescued TCF/LEF transcriptional activity [14]. These molecular alterations translated into reduced proliferation and migration in vitro and diminished tumor growth in vivo [51]. Similarly, RNA interference in MKN45 GC cells suppressed proliferation, adhesion, invasion, and lymphatic metastasis, in parallel with NFκB inactivation and decreased VEGF-C and MMP-9 expression [52], suggesting that CDH17 may orchestrate multiple pro-tumoral pathways beyond Wnt alone. Furthermore, this Wnt-centered regulation is not restricted to gastric models. In hepatocellular carcinoma (HCC), CDH17 knockdown inhibited proliferation of both primary and metastatic cell lines [53], an effect accompanied by cytoplasmic relocalization of β-catenin, downregulation of cyclin D1, and increased retinoblastoma protein levels. Notably, transcriptional control of *CDH17* by CDX2 and HNF1α further connected CDH17 expression to downstream *CCND1* activation [4], reinforcing the idea that CDH17 integrates differentiation programs with proliferative signaling. In colorectal cancer (CRC), the same signaling architecture appears conserved. CDH17 silencing in KM12SM and KM12C cells reduced viability and enhanced apoptosis, with increased caspase-3 and Bax and decreased Bcl-2 expression [54]. Interestingly, this pro-apoptotic shift was coupled with suppression of autophagy, as evidenced by reduced LC3 conversion. Pharmacological reactivation of autophagy partially rescued apoptosis, suggesting that CDH17 supports tumor cell survival through coordinated control of both Wnt signaling and autophagic flux. Similar findings in SW480 cells, where CDH17 inhibition reduced migration [55], further support a survival and motility-promoting function. Importantly, CDH17-mediated Wnt activation does not operate in isolation but intersects with integrin-dependent signaling pathways. Bartolomé et al. demonstrated that CDH17 physically interacts with α2β1 integrin in CRC cells, enhancing β1 integrin activation and talin recruitment, which in turn stimulates FAK, Ras, ERK, and JNK pathways, ultimately promoting cyclin D1 expression and proliferation [56]. This integrin activation critically depends on an exposed RGD motif located within the extracellular domain 6 of CDH17 [57], a structural feature that distinguishes CDH17 from classical cadherins. Additionally, mutation of this motif impaired tumor growth and liver homing in vivo, establishing a direct mechanistic link between CDH17-mediated adhesion and metastatic competence [57]. As emphasized in subsequent reviews from the same group, this RGD-mediated interaction functionally positions CDH17 as an unconventional cadherin with integrin-ligand properties, linking cell–cell adhesion to cell–matrix signaling and reinforcing its role in tumor progression [58]. Authors proposes that this CDH17–integrin axis may act as a signaling amplifier that cooperates with Wnt/β-catenin activation, thereby integrating adhesion cues with proliferative transcriptional programs. Together, these findings identify CDH17 not merely as an adhesion molecule, but as a molecular bridge between extracellular matrix interactions and intracellular oncogenic signaling.

The biological consequences of these integrative signaling effects become evident in in vivo models. In GC and PDAC, CDH17 suppression consistently reduced tumor growth, enhanced apoptotic signaling and chemotherapy sensitivity, often accompanied by decreased Akt phosphorylation and inactivation of the Ras/Raf/MEK/ERK cascade [59–61]. Conversely, CDH17 overexpression promoted xenograft growth, reinforcing its pro-survival role. These convergent findings across digestive tumor types suggest that CDH17 acts as a nodal amplifier of proliferative and anti-apoptotic pathways.

The role of CDH17 in tumor invasion and metastatic dissemination appears to be highly context-dependent and should be interpreted by clearly distinguishing between these two biologically distinct processes. In LoVo colorectal cancer (CRC) cells, CDH17 silencing increased MMP-2 and MMP-9 expression and enzymatic activity while reducing galectin-3 levels [62]. These findings indicate that CDH17 influences components of the extracellular matrix remodeling machinery. Given the established role of galectin-3 in regulating cell-matrix interactions, integrin signaling, and metalloproteinase activity in cancer progression [63, 64], modulation of galectin-3 by CDH17 may alter adhesive and invasive behavior at the local tumor level. In gastric cancer (GC), CDH17 and galectin-3 expressions were inversely correlated [65], further supporting the existence of a functional interplay between these molecules in regulating invasion-associated pathways. This inverse correlation also suggests that CDH17-mediated regulation of galectin-3 may vary according to tumor type and biological context, mirroring the context-dependent and phenotype-associated roles attributed to CDH17 itself during tumor progression.

However, these experimental observations relate primarily to local invasion and matrix remodeling rather than to the full metastatic cascade. An early and foundational contribution to the understanding of CDH17 in metastatic CRC was provided by a quantitative proteomic analysis comparing membrane-associated proteins between poorly metastatic and highly liver-metastatic KM12 CRC cell variants [66]. Focusing their analysis on the cell surface proteome, authors identified CDH17 as one of the most significantly upregulated proteins in highly metastatic KM12 cells. This differential expression was subsequently validated by immunohistochemical analyses in human tumor samples. Notably, CDH17 upregulation was observed in association with adhesion- and junction-related proteins, suggesting a reprogramming of epithelial adhesion networks in metastatic cells. More recently, single-cell transcriptomic analyses identified a CDH17-positive epithelial subpopulation enriched in tight junction components as a principal contributor to colorectal cancer liver metastases [67]. These CDH17⁺ cells displayed enhanced metastatic competence in vivo and defined a compact epithelial phenotype associated with collective migration. These concordant findings positioned CDH17 as a metastasis-associated surface protein in CRC. Additionally, the data derived from invasion assays and those derived from metastatic models reflect distinct biological contexts, suggesting that CDH17 may differentially influence local invasive capacity and metastatic colonization depending on tumor state and cellular phenotype.

This context-dependent behavior is also consistent with CDH17’s interactions within broader adhesion networks. Direct binding to p120-catenin modulates its localization and actin dynamics [68], while cooperation with the desmosomal cadherin DSC1 affects invasion in a phenotype-dependent manner. Together, these findings reinforce the concept that CDH17 contributes to tumor progression through modulation of adhesion and signaling programs whose impact varies across different stages of dissemination.

More recently, attention has shifted toward CDH17’s role in stemness and therapeutic resistance. The recent work by Bartolomé and colleagues provides an important mechanistic advance in metastatic CRC [69]. In metastatic CRC cell models, genetic silencing of CDH17 produced a marked reduction of the intestinal CSC marker LGR5, which was accompanied by inhibition of canonical Wnt/β-catenin signaling and downregulation of Wnt-driven pluripotency programs, notably MYC, resulting in reduced stemness properties. Moreover, treatment with CDH17 blocking antibodies phenocopied CDH17 loss, similarly decreasing LGR5 expression and Wnt pathway activity, supporting a functional CDH17-integrin axis upstream of LGR5/Wnt signaling. Beyond stemness, authors connected this pathway to drug resistance by showing that CDH17 depletion downregulated multiple drug-resistance–associated transporters, including the glutamine transporter SLC38A5, and increased sensitivity to 5-fluorouracil and irinotecan as well as to oxidative stress and anoïkis. Mechanistically, SLC38A5 silencing was required for CDH17-driven effects on survival and drug resistance, positioning SLC38A5 as a key downstream effector. Finally, pharmacological inhibition of SLC38A5 using amiloride increased chemosensitivity and improved survival in mouse metastasis models, providing a translational proof-of-concept for targeting this CDH17-LGR5/Wnt-MYC-SLC38A5 axis in metastatic CRC. Similarly, another study demonstrated that the CDH17-YAP/TAZ signaling axis plays a critical role in maintaining stemness properties and chemoresistance in a lung cancer circulating tumor cell model [49]. These observations suggest that CDH17 may extend its influence from proliferative signaling toward maintenance of a therapy-resistant tumor cell state. Additionally, these findings raise the possibility that CDH17-driven stemness programs may be influenced by the epithelial or mesenchymal status of tumor cells. In the lung cancer model, CDH17 upregulation was specifically observed in non-adherent, clustered circulating tumor cells displaying stem-like features [49], whereas the CRC study focused predominantly on epithelial metastatic cell lines [69]. Notably, experimental evidence in colorectal cancer has shown that CDH17 downregulation can exert opposite effects on migration and invasion depending on whether tumor cells display an epithelial or mesenchymal phenotype [68].

Taken together, these interconnected findings portray CDH17 not simply as an adhesion molecule but as a signaling hub that coordinates differentiation status, proliferative signaling, invasion dynamics, stemness, and therapeutic response in digestive cancers.

## Cadherin 17 as a diagnostic marker

CDH17 expression is largely restricted to intestinal epithelium under physiological conditions and is frequently maintained in gastrointestinal malignancies, particularly colorectal adenocarcinoma (CRC) [35, 70], where diffuse and strong expression is observed in the vast majority of cases. It is also reported in gastric cancer [71, 72], hepatocellular carcinoma [53, 73, 74], pancreatic ductal adenocarcinoma [36], particularly in the exocrine-like subtype of PDAC [75], and in digestive neuroendocrine tumors (NETs) [76–78], as well as in Barrett’s esophagus–associated carcinoma [79] and Crohn disease–associated small bowel neoplasms [80]. In this context, CDH17 primarily represents a lineage-associated differentiation marker reflecting intestinal-type epithelial programs rather than a tumor-specific biomarker. Table 1 summarizes the main immunohistochemical (IHC) studies evaluating CDH17 expression in digestive tumors.

**Table 1:** Diagnostic immunohistochemical studies using CDH17 in digestive tumors

Organ	Tumor	Cases number	Associated studied markers	Results & Performance (CDH17)	Reference
Colorectal cancer					
Lung metastasis	Metastasis	275	GPA33 MUC2 MUC6 SATB2 SMAD4	Sensitivity: 99%	Malmros et al. 2024 [81]
Colorectal tissue	Primitive & metastasis	65	CDX2	Sensitivity: 100% Specificity: 50%	Abouelkhair et al. 2021 [82]
Lung metastasis	Metastasis	27	SATB2 CK7 CK20 CDX2	Combination CDH17/SATB2 Sensitivity: 76,9% Specificity: 100%	Bian et al. 2017 [83]
Cytology samples	Metastasis (EUS-FNA)	43	SATB2 CK20 CDX-2	Sensitivity: 97,6% Specificity: 83,8%	Brandler et al. 2015 [140]
Lung metastasis and lymph nodes	Primitive & metastasis	44	CDX2	Concordance metastases: 74,1% Concordance nodes: 84,3%	Park et al. 2011 [70]
Other digestive tumors					
Cytology samples	Metastasis (ascite, pleural, pericardic effusion)	180	CDX2	Sensitivity: 80,7% Specificity: 85,2%	Ma et al. 2019 [141]
Gastric	Primitive & metastasis	175	CDX2 CK20	Positivity CDH17 > CDX2 & CK20	Altree-Tacha et al. 2017 [84]
Gastric	Primitive & metastasis	98	CLDN18	Positivity: 89,3%	Matsusaka et al. 2016 [142]
Digestive NET	Primitive & metastasis	165	CDX2 TTF1	Sensitivity CDH17 > CDX2	Snow et al. 2015 [76]
Pancreas	Primitive (PDAC)	135	CDX2	Positivity: 70%	Morimatsu et al. 2012 [85]
Digestive tumors	Primitive	777	CDX2	Positivity CDH17 > CDX2	Panarelli et al. 2012 [86]
Esophagus	Primitive	90	CDX2	Positivity: 70%	Weimann et al. 2010 [143]
Digestive tumor	Primitive	528	CK7	CDH17+/CK7- identified 97% of CRC CDH17+/CK7 + identified 86% of other digestive tumors	Min-Cheng et al. 2008 [144]
Liver	Primitive (HCC) and healthy tissues	13	/	CDH17 overexpression in HCC compared to healthy tissue (72%)	Wong et al. 2003 [73]

*HCC* Hepatocellular carcinoma, *NET* Neuroendocrin tumor, *PanNET* pancreatic neuroendocrine tumors, *PDAC* Pancreatic ductal adenocarcinoma, *EUS-FNA* Endoscopic Ultrasound-Guided Fine Needle Aspiration

In colorectal cancer, CDH17 expression is highly concordant between primary tumors and corresponding metastases, including lung and lymph node lesions [70], supporting its usefulness in determining intestinal origin. Several comparative studies have evaluated CDH17 alongside CDX2 [82, 83], the current reference marker for intestinal differentiation. Overall, CDH17 demonstrates sensitivity comparable to CDX2 for identifying colorectal metastases, although specificity remains moderate. For example, Abouelkhair et al. [82] reported similar diagnostic performance between CDH17 and CDX2 in a retrospective cohort of colorectal and non-colorectal adenocarcinomas. Likewise, Bian et al. showed that combining CDH17 with SATB2 improved specificity in distinguishing pulmonary enteric adenocarcinoma from metastatic CRC [83]. In a large cohort of 275 patients with pulmonary metastases from CRC, Malmros et al. [81] reported that CDH17 and CDX2 exhibited the highest sensitivity (99% for both), whereas SATB2 and CK20 offered greater specificity. However, no single marker or combination completely discriminated metastatic CRC from primary lung adenocarcinoma. Importantly, CDH17 and CDX2 expression patterns are frequently correlated, consistent with their shared association with intestinal lineage specification.

In other digestive tumors, expression patterns are more heterogeneous. Early studies suggested CDH17 overexpression in hepatocellular carcinoma [73], but subsequent larger series reported substantially lower positivity rates [87], highlighting inter-cohort variability. A comprehensive IHC study by Panarelli et al. [86] demonstrated frequent coexpression of CDH17 and CDX2 in colorectal, gastric, and esophageal adenocarcinomas, with rare discordant profiles. In pancreatic adenocarcinoma and cholangiocarcinoma, CDH17 positivity exceeded that of CDX2 [86], again reflecting differentiation patterns rather than strict tumor-type specificity. In well-differentiated digestive NETs, CDH17 expression varies according to anatomical origin [76–78], with higher positivity in midgut and hindgut tumors, and generally mirrors primary tumor expression in metastatic lesions.

Importantly, CDH17 expression has also been described in certain non-gastrointestinal tumors, including subsets of pancreatic and bronchial NETs [86]. In many of these contexts, CDH17 positivity appears to correlate with intestinal-type differentiation or mucinous features rather than anatomical site of origin. These observations underscore the need to interpret CDH17 staining within the framework of tissue lineage and differentiation status.

Beyond IHC, exploratory studies have evaluated CDH17 as a circulating or secreted diagnostic biomarker. Plasma ELISA analyses in gastric cancer showed limited diagnostic performance, with moderate sensitivity and specificity and no clear discrimination in early stages [38]. Proteomic investigations have also identified CDH17 ectodomain shedding in xenograft models, suggesting theoretical potential for circulating biomarker development [88]. However, these approaches remain preliminary and currently lack robust experimental and clinical validations.

Taken together, current evidence supports CDH17 primarily as a marker of intestinal differentiation that may assist in determining tumor origin in selected diagnostic settings. Nevertheless, available data remain largely heterogeneous, and do not yet establish CDH17 as a standalone robust diagnostic or prognostic biomarker.

## Cadherin 17 as a prognosis marker

Beyond its role in tumor diagnosis, the prognostic significance of CDH17 expression represents the most extensively studied aspect of this marker. Numerous studies have demonstrated significant correlations between CDH17 expression levels and clinicopathological parameters, as well as patient outcomes, across a wide range of digestive malignancies, including colorectal, gastric, hepatic, and pancreatic cancers. However, reported associations are frequently heterogeneous and occasionally contradictory, highlighting the context-dependent role of CDH17 during tumor progression and indicating that its prognostic utility remains to be clearly defined. Table 2 summarizes the main studies assessing the prognostic value of CDH17 expression in digestive cancers.

**Table 2:** Prognostic significance of CDH17 expression in digestive cancers

Study / cohort information’s	Patients (*n*)	CDH17 evaluation method	Prognostic association	References
Colorectal cancer				
Digital IHC platform with image-based scoring (‘Membrane score’)	150	IHC (Digital membrane score)	High CDH17 score predicts poorer OS and RFS; associated with advanced stage and distant metastasis	Ng L et al. 2024 [89]
Population-based IHC study	2351	IHC	Low CDH17 expression associated with advanced stage, vascular invasion, right-sided location, MSI; suggests tumor-suppressive association	Jacobsen F et al. 2024 [34]
Retrospective cohort	45	IHC	Reduced CDH17 expression associated with high grade, lymphatic invasion, LN metastasis, advanced pTNM stage	Takamura M et al. 2004 [37]
Retrospective cohort	207	IHC	Reduced CDH17 expression correlated with dedifferentiation and independently predicted worse OS	Kwak J-M et al. 2007 [38]
Cohort with lymphovascular invasion (tumor emboli analysis)	84	IHC	High CDH17 expression in tumor emboli correlated with advanced T stage and poorer OS (dual role with differentiation context)	Ianole V et al. 2025 [145]
Serum biomarker study (CRC vs. healthy controls)	110 CRC + 90 controls	ELISA (Serum CDH17)	Serum CDH17 concentrations downregulated in CRC and associated with location, lymphatic metastasis, invasion depth, differentiation and stage	Wang X et al. 2020 [90]
Gastric cancer				
Retrospective cohort	94	IHC	CDH17 positivity more frequent in intestinal-type and advanced-stage GC and associated with poorer prognosis	Ito R et al. 2005 [72]
Retrospective cohort	166	IHC	CDH17 positivity associated with deeper invasion, LN metastasis and advanced stage, and linked to poorer prognosis	Ge J et al. 2008 [91]
Tumor vs. adjacent tissue cohort	204	IHC	CDH17 positivity more frequent in GC vs. adjacent tissue, and associated with size, Lauren type, stage, and distant metastasis	Tu L et al. 2016 [92]
Meta-analysis	2,120	IHC (pooled studies)	Elevated CDH17 expression associated with advanced features (TNM stage, differentiation, invasion depth, LNM)	Long Z-W et al. 2015 [93]
Meta-analysis	—	IHC (pooled studies)	Association of CDH17 positive expression with deeper invasion; other associations inconsistent across studies	Meng W et al. 2015 [94]
Metaplasia-associated biomarker panel study	450	IHC	Reduced intestinal differentiation markers including CDH17 associated with undifferentiated histology, advanced stage and worse outcomes	Suh Y-S et al. 2012 [39]
Multiplex IHC panel with TCGA RNA-seq validation	329	Multiplex IHC + RNA-seq validation	Low/absent CDX2 program including CDH17 identifies subgroup with poor overall survival	Lopes N et al. 2020 [95]
Biopsy and resection cohort (LNM prediction)	208	IHC (biopsy and surgical specimens)	Reduced CDH17 expression linked to progression and LNM with CDH17 status independently predicts LNM	Park S-S et al. 2007 [96]
Node-negative (pN0) cohort (LN micrometastasis)	191	IHC	CDH17 positivity associated with LN micrometastasis; and prognostic impact in selected subgroups	Wang J et al. 2012 [97]
Gene expression cohort	47	mRNA (as reported)	Higher CDH17 mRNA expression associated with distant metastasis and lymphatic invasion	Ryu KH et al. 2012 [98]
IM/SPEM transcriptomics with GC tissue validation	78	Gene expression + tissue validation	CDH17 negative expression associated with poorer survival in stage I / node-negative GC	Lee H-J et al. 2010 [99]
Hepatocellular carcinoma				
HBV-positive HCC cohort	255	IHC + Western blot (subset)	CDH17 overexpression associated with microvascular invasion, recurrence and reduced OS	Ding Z-B et al. 2009 [74]
CK19-postive HCC cohort	114	IHC	CDH17 expression associated with CK19 expression, and anindependent predictor of DFS	Lee C-W et al. 2018 [100]
Large-scale intestinal differentiation marker study in HCC	202	IHC	CDH17 positivity in a minority of cases and associated with high grade and elevated AFP	Kmeid M et al. 2021 [87]
Splice variant study (exon 7 skipping)	50 tumors + 8 controls	Splice variant analysis (as reported)	*CDH17* exon 7–skipping variant detection associated with recurrence, venous infiltration and decreased OS	Wang XQ et al. 2005 [101]
Case–control genetics study	164 tumors + 99 cirrhosis + 293 controls	Genotyping (SNPs / haplotype)	*CDH17* haplotype associated with increase HCC susceptibility	Wang XQ et al. 2006 [102]
Biliary tumors				
Intrahepatic cholangiocarcinoma cohort	34	IHC	Absence of CDH17 expression associated with dedifferentiation, vascular invasion and shorter survival	Takamura M et al. 2010 [103]
Cholangiocarcinoma cohort	180	IHC	High CDH17 expression associated with LNM and worse OS/RFS	Zheng B-H et al. 2021 [16]
Pancreatic tumors				
PDAC cohort	102	IHC	High CDH17 in well-differentiated tumors; improved OS; independent prognostic factor	Takamura M et al. 2003 [36]
PDAC proteomics study (short vs. long survival)	19	Proteomics	CDH17 enriched in favorable survival group (exploratory; not retained in final candidate set)	Hu D et al. 2018 [104]
IPMN cohort	135	IHC + RT-qPCR	Higher CDH17 in intestinal-type IPMN; increases with grade and correlates with Ki67	Morimatsu K et al. 2012 [85]

*CRC* colorectal cancer, *GC* gastric cancer, *HCC* hepatocellular carcinoma, *PDAC* pancreatic ductal adenocarcinoma, *IPMN* intraductal papillary mucinous neoplasm, *iCCA* intrahepatic cholangiocarcinoma, *LNM* lymph node metastasis, *LNMM* lymph node micrometastasis, *OS* overall survival, *RFS* recurrence-free survival, *DFS* disease-free survival, *MSI* microsatellite instability, *AFP* alpha-fetoprotein, *IHC* immunohistochemistry, *RT-qPCR* reverse transcription quantitative polymerase chain reaction, *TCGA* The Cancer Genome Atlas

### Colorectal cancer

The prognostic significance of CDH17 in colorectal cancer (CRC) remains controversial and appears highly stage- and context-dependent. While CDH17 is physiologically expressed in intestinal epithelium, its expression dynamics during tumor progression are complex.

Several studies focusing on primary CRC cohorts have reported that reduced CDH17 expression correlates with tumor dedifferentiation, vascular and lymphatic invasion, advanced stage, and worse overall survival [34, 37, 38, 90, 105]. In these series, CDH17 loss was frequently associated with high-grade tumors and features of local aggressiveness, suggesting that downregulation may reflect epithelial destabilization and increased invasive potential. This association with aggressive clinicopathological features is supported by experimental data showing that CDH17 regulates invasion-related pathways, notably through modulation of galectin-3 and matrix metalloproteinase activity, thereby influencing extracellular matrix remodeling and cell–matrix interactions [61, 62]. Supporting this, serum CDH17 levels were reduced in CRC patients and associated with advanced clinicopathological parameters [90]. Epigenetic analyses further identified CDH17 promoter hypermethylation as an independent predictor of relapse in stage II colon cancer [105], reinforcing the association between CDH17 loss and early disease progression.

In contrast, studies specifically addressing metastatic CRC have reported opposite findings. Increased CDH17 expression has been observed in distant metastases compared to primary tumors [106]. Notably, an immunohistochemical analysis of CDH17 expression in 119 metastatic CRC samples demonstrated that high CDH17 expression was significantly associated with poor overall survival [56]. Similarly, digital image–based analyses in advanced CRC cohorts linked high CDH17 expression to distant metastasis and unfavorable outcomes [89]. Importantly, these 2 studies primarily included stage IV or metastatic disease, in contrast to studies focusing on lymph node involvement or earlier stages.

Taken together, these data suggest a possible dual role for CDH17 during CRC progression. Early downregulation of CDH17 may facilitate epithelial destabilization and local invasion, whereas re-expression or maintained expression at later stages may contribute to metastatic colonization and outgrowth, consistent with a model of epithelial plasticity during dissemination.

These observations point toward a stage-related biological role rather than a simple high-versus-low prognostic model.

### Gastric cancer

As in CRC, the prognostic significance of CDH17 in GC is heterogeneous and appears strongly influenced by tumor subtype, stage distribution, and the clinical endpoints evaluated. CDH17 expression in GC closely reflects intestinal differentiation programs, which complicates interpretation when cohorts differ in Lauren classification or biological composition.

Several studies conducted in mixed-stage surgical cohorts have reported that CDH17 overexpression correlates with advanced disease and unfavorable outcomes. Ito et al. [72] analyzed 94 GC cases and observed CDH17 positivity in 67% of tumors, with higher expression in intestinal-type carcinomas and advanced-stage disease, and significantly poorer prognosis in CDH17-positive patients. Similarly, Ge et al. [91] reported CDH17 positivity in 60.8% of 166 GC cases, with significant associations with deeper invasion, lymph node metastasis, advanced TNM stage, and worse survival. Tu et al. [92], in a cohort of 204 paired GC and adjacent tissues, confirmed increased CDH17 expression in tumors and its correlation with tumor size, Lauren classification, stage, and distant metastasis. Meta-analyses further explored these associations. Long et al. [93], analyzing compiled data from over 2,000 patients, reported that CDH17 overexpression was significantly associated with advanced stage, deeper invasion, poor differentiation, and lymph node metastasis. However, another meta-analysis confirmed the association with deeper invasion but did not observe consistent correlations with TNM stage or histological grade, underscoring substantial inter-study heterogeneity [94]. Taken together, these data suggest that in broadly mixed GC populations, CDH17 overexpression may accompany advanced tumor progression and adverse outcomes, although variability in cohort composition and scoring methodologies likely contributes to inconsistent findings.

In contrast, other studies emphasize the prognostic significance of intestinal differentiation programs rather than absolute CDH17 expression levels. In a large cohort of 450 GC patients, Suh et al.. demonstrated that reduced expression of a panel of metaplasia-associated markers, including CDH17, was associated with undifferentiated histology, larger tumor size, advanced stage, and poorer survival [39]. Similarly, another study showed that low or absent expression of CDX2 and downstream targets such as CDH17 defined a subgroup with particularly poor overall survival in a multiplex IHC study validated by TCGA RNA-seq data [95]. Lee et al. also reported that CDH17 loss predicted adverse prognosis, particularly in early-stage GC [99]. These findings indicate that loss of intestinal differentiation features may reflect tumor dedifferentiation and biological aggressiveness. In this setting, CDH17 negativity appears to mark collapse of lineage identity rather than indolent behavior.

Studies specifically addressing lymph node involvement further illustrate, like in CRC, the context-dependent nature of CDH17 associations. Park et al. reported that reduced CDH17 expression independently predicted lymph node metastasis, suggesting that loss of epithelial adhesion features may facilitate early dissemination [96]. Conversely, another research group found that CDH17 positivity was associated with lymph node micrometastasis and worse survival in a cohort restricted to pN0 patients [97]. Elevated *CDH17* mRNA levels in diagnosis biopsies were also associated with lymphatic invasion and distant metastasis in an independent study [98]. These discrepancies likely reflect differences in patient selection, biological stage assessed, and tumor phenotype, indicating that CDH17 expression may have distinct implications depending on whether early dissemination or established metastatic growth is evaluated.

In consequence, in GC, CDH17 appears more closely linked to lineage identity and tumor evolution than to a fixed prognostic category.

### Liver and pancreatic cancers

Aberrant CDH17 expression has been described in primary liver and pancreatic malignancies, although its prognostic significance, like for CRC and GC, remains context-dependent and influenced by tumor subtype and differentiation status.

In HCC, several studies support an association between CDH17 overexpression and aggressive tumor features. In a large cohort of hepatitis B virus–positive HCC patients (*n* = 255), CDH17 overexpression correlated with microvascular invasion, higher recurrence rates, and reduced overall survival [74]. Functional experiments in highly invasive HCC cell lines further showed that CDH17 silencing reduced migration, adhesion, and invasion, supporting a pro-invasive role [74]. Independent studies linked CDH17 expression to CK19 positivity, a marker of poor prognosis and progenitor-like phenotype, and demonstrated that CDH17 independently predicted reduced disease-free survival [100]. In a separate cohort of 202 HCC cases, CDH17 expression, although present in only 10.3% of tumors, was significantly associated with high tumor grade and elevated AFP levels, defining an intestinal-like aggressive subset enriched in CK19-positive tumors [87].

Genetic alterations further support a role for CDH17 in HCC progression. A splice variant lacking exon 7 was detected in approximately 50% of tumors and was associated with venous invasion, recurrence, and poor survival [101]. Specific CDH17 single-nucleotide polymorphisms were enriched in HCC patients compared with cirrhotic controls, suggesting a potential germline susceptibility component [102]. Although direct functional evidence in HCC remains limited, parallels with colorectal cancer indicate that CDH17 variants may impair adhesive function and promote tumor dissemination [107, 108].

In intrahepatic cholangiocarcinoma (iCCA), findings are more heterogeneous. One study associated loss of CDH17 expression with dedifferentiation, vascular invasion, and shorter survival [103], whereas another reported that CDH17 overexpression correlated with lymph node metastasis and poorer postoperative survival, outperforming CA19-9 in prognostic models [109]. CDH17 expression was also detected in high-grade biliary intraepithelial lesions (BilIN-2/3), supporting its association with advanced biliary disease [110].

In pancreatic tumors, CDH17 expression appears primarily linked to epithelial differentiation. In pancreatic ductal adenocarcinoma (PDAC), strong CDH17 expression was observed in well-differentiated tumors and was largely absent in poorly differentiated or advanced-stage cancers [36]. High CDH17 expression was independently associated with improved overall survival, with preserved epithelial differentiation conferring a more favorable prognosis [36]. Proteomic analyses comparing PDAC patients with markedly different postoperative survival identified CDH17 among proteins enriched in long-term survivors; however, subsequent refinement of the prognostic signature excluded CDH17, limiting its independent predictive value [104].

Regarding intraductal papillary mucinous neoplasms (IPMN) preneoplastic pancreatic lesions, CDH17 expression was significantly higher in intestinal-type lesions and increased progressively from low-grade dysplasia to invasive carcinoma, correlating with proliferative activity [85]. These findings tend to indicate that CDH17 participates in the intestinal differentiation–associated carcinogenic pathway in pancreatic neoplasia.

Overall, in liver and pancreatic malignancies, CDH17 expression appears closely linked to tumor lineage and differentiation programs. In HCC, its overexpression defines a subset enriched for invasive and progenitor-like features, whereas in PDAC and intestinal-type pancreatic neoplasms, preserved expression is mainly associated with maintained epithelial differentiation.

### Non-digestive tumors

Aberrant neoexpression of CDH17 has also been reported in non-digestive malignancies, particularly ovarian and lung cancers, where it is generally associated with aggressive tumor behavior and poor clinical outcomes. In epithelial ovarian cancer (EOC), analysis of 182 cases showed significant CDH17 upregulation concomitant with CDX2 downregulation [43]. High CDH17 expression correlated with advanced stage, high tumor grade, and reduced overall survival, with the CDH17-high/CDX2-low profile identifying the poorest prognosis group. Multivariate analysis confirmed the combined CDH17/CDX2 expression status as an independent prognostic factor [43]. Interestingly, this dissociation between CDH17 and CDX2 is unusual compared with digestive malignancies, in which CDH17 expression typically parallels CDX2-driven intestinal differentiation programs, and suggests that CDH17 expression may be regulated independently of canonical intestinal lineage transcriptional control in EOC.

In lung adenocarcinoma (LUAD), multiple transcriptomic studies converged on CDH17 as a marker of poor prognosis. Using The Cancer Genome Atlas datasets, Xie et al. identified a 14-gene junctional signature, including CDH17, significantly associated with unfavorable clinical outcomes [46]. A risk score derived from this signature, driven in part by elevated CDH17 expression, stratified patients by overall survival and was associated with higher tumor mutational burden. Functional enrichment analyses indicated that the high-risk group was characterized by activation of cell proliferation and immune regulation pathways [46]. Consistently, exosome-based transcriptomic analyses identified CDH17 as part of a 12-gene prognostic model derived from exosome-associated differentially expressed genes (ExoDEGs), which effectively stratified LUAD patients into high- and low-risk survival groups [48]. High-risk patients exhibited increased CDH17 expression and features of an immunosuppressive tumor microenvironment, including reduced immune activity, suggesting a role for CDH17 in immune evasion [48]. These findings were further supported by an independent TCGA-based study that identified CDH17 among five immune-related genes forming a prognostic model associated with poor outcomes in LUAD [47], and in line with previously cited study highlighting that high CDH17 expression in LUAD circulating tumor cells confers stemness and chemoresistant properties [49].

Overall, the prognostic significance of CDH17 in digestive neoplasm does not follow a simple high-versus-low model. Its expression primarily reflects tumor differentiation status, lineage identity, and stage-specific biological context. While CDH17 may hold prognostic value within selected subgroups, current evidence does not support its use as a standalone biomarker, and further stage- and subtype-stratified studies are warranted.

## Therapeutic strategies targeting CDH17

CDH17 has emerged as a compelling therapeutic target in digestive malignancies due to its restricted expression in normal colorectal epithelium and frequent overexpression in various GI tumors, allowing the development of targeted therapies with potentially minimized off-target toxicity. Table 3 provides an overview of key preclinical studies investigating CDH17-targeted drugs or agents in therapeutic settings.

**Table 3:** CDH17-targeted therapeutic strategies in digestive cancers

Target Cancer Types	Therapeutic modality	Target / Payload / Mechanism	Model(s)	References
Digestive tumors	Bispecific ADC	TRAILR2×CDH17-ADC / irinotecan	Cell lines, CDX, PDX	Garcia Martinez et al. 2021 [111]
Digestive tumors	CAR-NK cells	CDH17-targeted CAR-NK	Cell lines, CDX	Zheng et al. 2022 [122]
Digestive tumors and NETs	CAR-T cells	CDH17 targeted CAR-T	Cell lines, CDX, autochthonous models	Feng et al. 2022 [112]
Digestive tumors	Biohybrid bacteria-mediated photothermal therapy	CDH17 nanobody coupled with MG1655 bacteria and photosensitizer croconium	Syngeneic mouse models	Xu et al. 2024 [113]
Digestive tumors	ADC	CDH17-ADC with topoisomerase inhibitor MF-6	Cell lines, CDX, PDX, NHP	Wang et al. 2025 [114]
Colorectal cancer	Nanobody–immunotoxin	CDH17-targeted nanobody-mediated delivery of PE38 toxin	Cell lines, CDX	Ding et al. 2024 [115]
Colorectal cancer	CAR-T cells	CDH17-targeted CAR-T	Cell lines, CDX	Greco et al. 2025 [116]
Colorectal cancer	Bispecific ADC	pCAD×CDH17 / cytotoxic agent MMAE	Cell lines, CDX	Synan et al. 2025 [117]
Colorectal cancer	Bispecific ADC	GUCY2C×CDH17 / ferroptosis inducer RSL3	Cell lines	Zhang et al. 2025 [123]
Gastric cancer	Immunotoxin	Multi-epitope CDH17 targeting linked to saporin	Cell lines	Kusano-Arai et al. 2018 [118]
Gastric cancer	Engineered extracellular vesicles	CDH17-targeted EVs loaded with RRx-001	Cell lines; PDX	Peng et al. 2022 [125]
Gastric cancer	Nanobody-immunotoxin	CDH17-targeted nanobody-mediated delivery of PE38 toxin	Cell lines, CDX, PDX	Ma et al. 2022 [146]
Gastric cancer	Radiotheranostic antibody	CDH17-antibody coupled with radionuclide ^177^Lu	Xenograft models	Mao et al. 2025 [119]

*ADC* Antibody–drug conjugate, *CAR-T* Chimeric antigen receptor T cells, *CAR-NK* Chimeric antigen receptor natural killer cells, *CDH17* Cadherin 17, *CDX* Cell line–derived xenograft, *EVs* Extracellular vesicles, *GC* Gastric cancer, *GPX4* Glutathione peroxidase 4, *ICG* Indocyanine green, *MMAE* Monomethyl auristatin E, *mAb* Monoclonal antibody, *NHP* Non-human primates, *PDX* Patient-derived xenograft, *RRx-001* Dinitroazetidine derivative radiosensitizer

In addition to its biological functions, the translational potential of CDH17 has previously been highlighted by the *Casal* group, particularly in the context of integrin blockade and antibody-based strategies [120]. Of particular interest is the possibility of targeting the exposed RGD motif located within the extracellular domain of CDH17, thereby disrupting its interaction with α2β1 integrins and subsequent activation of downstream oncogenic signaling pathways. This structural feature provides a clear mechanistic rationale for the development of selective therapeutic strategies aimed at inhibiting integrin-dependent tumor growth while taking advantage of the restricted physiological expression of CDH17 in digestive tissues. The same research group took a pivotal step toward therapeutic validation by establishing CDH17 as a functionally actionable oncogenic driver in colorectal cancer [121]. In this study, monoclonal antibodies targeting CDH17 disrupted the CDH17–integrin interaction, resulting in reduced proliferative signaling, and inhibition of tumor growth. Importantly, antibody-mediated targeting significantly decreased liver metastasis formation in vivo without major toxicity, providing a robust preclinical proof-of-concept that CDH17 itself represents a therapeutically relevant molecular vulnerability with a potentially favorable safety profile.

Building on this foundational work, subsequent strategies not only aimed to block CDH17-driven signaling, but also leveraged its tumor-restricted expression as a selective delivery platform for therapeutic payloads, further expanding the translational landscape of CDH17-directed interventions.

First, several recent studies have explored CDH17-targeted immunotherapy strategies for therapeutic intervention in digestive cancers. Notably, Feng et al. engineered CDH17-specific chimeric antigen receptor (CAR) T cells and demonstrated their robust antitumor efficacy against gastrointestinal cancers (GICs) and NETs in multiple mouse xenograft models [112]. These VHH1-CAR T cells efficiently eradicated CDH17-expressing gastric, pancreatic, and colorectal tumors while sparing normal tissues. Importantly, no toxicity was observed in the normal colorectal mucosa, which the authors attributed to the basolateral confinement of CDH17 expression in healthy epithelium, likely limiting CAR-T cell access and enhancing treatment specificity [112]. In the same way, a recent study reports the development of engineered nanobody-based chimeric antigen receptor natural killer (CAR-NK) cells targeting CDH17 for the treatment of GICs [122]. They further demonstrated that CDH17-CAR-NK cells effectively eliminate GIC cells both in vitro and in vivo, using cell line-derived and patient-derived xenograft mouse models. Finally, a very recent study conducted by an Italian team identifies CDH17 as a promising antigen for CAR T cell therapy in CRC liver metastases [116]. Various CDH17 CAR constructs, differing in binding and spacer regions, were evaluated in preclinical mouse models. While binding domain influenced in vitro efficacy, spacer composition shaped in vivo dynamics. In a CRC liver xenograft model, CDH17 CAR-T cells effectively inhibited tumor growth via both systemic and locoregional delivery [116]. Together, these findings support systemic administration of CDH17 CAR T cells as a novel, promising, and potentially safe immunotherapeutic strategy, warranting clinical investigation.

In a parallel effort to enhance selectivity and reduce toxicity, CDH17 bispecific antibody-drug conjugates (ADC) are currently being developed. Using a dual-targeting strategy, Synan et al.. developed an ADC targeting both CDH17 and pCAD [117], in order to restrict cytotoxicity to tumor cells co-expressing both antigens. Preclinical models of CRC demonstrated potent tumor growth inhibition with the pCAD × CDH17 bispecific ADC, while normal tissues remained largely unaffected. Bulk and single-cell RNA sequencing, corroborated by IHC, confirmed high co-expression of the target antigens in malignant tissues and minimal expression in healthy counterparts, suggesting a favorable safety profile. Another team also chose this dual specificity strategy to generate an ADC targeting CDH17 and GUCY2C, and conjugated with the GPX4 inhibitor RSL3, thereby inducing ferroptosis, an iron-dependent form of cell death driven by lipid peroxidation [123]. By triggering ferroptosis, the BsADC achieved superior binding, internalization, and inhibition of tumor cell proliferation compared with single-target ADCs, while maintaining a favorable safety profile in mice [124]. In the same way, BI 905,711, a tetravalent bispecific antibody targeting both TRAILR2 and CDH17, was developed to specifically target colorectal tumors [111]. Preclinical experiments demonstrated an effective induction of apoptosis in a broad range of CDH17-positive colorectal cancer cell lines, while the potency was reduced by more than 1,000-fold in CDH17-negative cells. Further in vivo assays highlighted significant tumor regressions in xenograft models, with enhanced efficacy observed when combined with irinotecan chemotherapy. Interestingly, BI905711 also showed activity in models with heterogeneous CDH17 expression, via both cis (same cell) and trans (adjacent cell) mechanisms [111]. A Phase I study (NCT04137289) is currently ongoing on colorectal and other CDH17-positive tumors. Finally, Rui et al. very recently reported the development of 7MW4911, a CDH17-targeted ADC carrying the topoisomerase inhibitor MF-6 [114]. 7MW4911 demonstrated high specificity for CDH17-expressing tumors and potent antitumor activity in multiple preclinical models, including patient-derived xenografts (PDXs) with diverse genetic backgrounds, achieving tumor growth inhibition rates between 71% and 99%. The compound also showed favorable pharmacokinetics and a high non-toxic threshold (> 20 mg/kg) in cynomolgus monkeys [114].

Using the AGS gastric cancer cell model, another team developed multiple anti-CDH17 monoclonal antibodies (mAb) to recognize distinct epitopes on the extracellular region of CDH17 [118]. These cells were further classified into subpopulations with low, medium, and high CDH17 surface expression (AGSlow, AGSmed, AGShigh). When conjugated to saporin, a ribosome-inactivating toxin, individual mAbs showed strong cytotoxicity in AGShigh cells but limited effects on AGSlow cells. However, a cocktail of three CDH17-targeted immunotoxins, each targeting a different epitope, exhibited synergistic cytotoxicity even in low-expressing AGSlow cells [118]. This synergy demonstrates the potential of multi-epitope strategies to overcome tumor heterogeneity and improve the efficacy of antibody-based antitumoral therapeutic strategies.

Beyond conventional antibody- and cell-based CDH17-targeted strategies, more original therapeutic approaches have been explored. Notably, a bacterial delivery platform using CDH17-specific nanobodies was developed for targeting digestive tumors [113]. An engineered biohybrid E. coli MG1655 strain, incorporating a CDH17 nanobody and conjugated to a croconium photosensitizer, selectively accumulated in CDH17-positive gastric, colorectal, and pancreatic tumors, leading to significant antitumor efficacy through photothermal effects and immune activation across multiple in vivo models. In addition, CDH17 has also been investigated as a targeting vector for radionuclide-based theranostic strategies. In a recent study, anti-CDH17 nanobodies were engineered in two forms: a polyhistidine-tagged variant (VHH) and a pharmacokinetically enhanced version fused with an albumin-binding domain (VHH-ABD) [119]. Both constructs were radiolabeled with lutetium-177 (^177Lu). In vivo experiments using MKN-45 gastric cancer xenografts demonstrated that [^177Lu]Lu-VHH-ABD displayed prolonged systemic retention and superior tumor accumulation compared to [^177Lu]Lu-VHH, with a significant tumor growth suppression and minimal systemic toxicity. Additionally, both radiolabeled agents facilitated high-resolution imaging of CDH17-positive tumors via SPECT/CT, highlighting their dual diagnostic and therapeutic potential [119]. A similar nanobody-based strategy was conducted by *Ding et al.*, with the synthesis of a CDH17-targeting immunotoxin (E8-Nb-PE38) [115]. This construct selectively targeted CDH17-expressing CRC cells and induced marked tumor growth inhibition in multiple xenografted models. Furthermore, combining E8-Nb-PE38 with 5-fluorouracil (5-FU) yielded synergistic antitumor effects and extended animal survival. In parallel and for diagnostic applications, the authors generated a near-infrared (NIR) fluorescent probe, which enabled high-contrast NIR-II imaging of CRC lesions, ultimately facilitating image-guided tumor resection [115].

Finally, a study introduces an innovative delivery system based on extracellular vesicles (EVs) functionalized with nanobodies targeting CDH17, specifically designed for GC therapy [125]. EVs derived from HEK-293 cells were engineered to display CDH17 nanobodies and loaded with either the imaging dye indocyanine green (ICG), the anti-cancer agent RRx-001 (a CD47–SIRPα axis inhibitor), or both. In CDH17-positive tumor models, ICG-loaded EVs enabled rapid tumor imaging and induced a strong photothermal therapy effect upon irradiation. RRx-001-loaded EVs significantly suppressed tumor growth, and dual-loaded EVs (ICG + RRx-001) achieved maximal anti-tumor efficacy after a single injection in both cell line- and PDXs models [125]. All these dual-function agents further reinforce the clinical potential of CDH17 as a target for both therapeutic and diagnostic applications in GI. Collectively, these studies underscore the growing relevance of CDH17 as a multifaceted target for innovative therapeutic strategies in digestive malignancies, encompassing immunotherapy, targeted drug delivery, and image-guided surgery.

In line with these preclinical findings, the translational relevance of CDH17 has recently been explored in the clinical setting. Several CDH17-targeted therapeutic strategies have now entered early-phase clinical development, aiming to translate the therapeutic properties of this antigen into patient benefit. As summarized in Table 4, ongoing clinical trials predominantly involve CAR-T therapies, bispecific antibodies, and ADCs, evaluated in patients with advanced or refractory CDH17-expressing solid tumors. Most of these studies are first-in-human or phase I trials primarily focused on safety, feasibility, and preliminary antitumor activity, marking the initial steps toward clinical validation of CDH17 as a therapeutic target.

**Table 4:** Clinical trials targeting *CDH17*

NCT number	Agent / Therapy	Modality	Target Cancer Types	Dual Targeting	Phase	Status	Clinical results
NCT06055439	CHM-2101	Anti-CDH17 CAR-T	GI cancers, colorectal, gastric, NETs	No	Phase I/II	Recruiting	Ongoing – no public clinical results
NCT06501183	Anti-CDH17 CAR-T	CAR-T	CDH17 + solid tumors	No	Phase I	Recruiting	Ongoing – no public clinical results
NCT06820424	Anti-CDH17 CAR-T	CAR-T	CDH17 + solid tumors	No	Phase I	Recruiting	Ongoing – no public clinical results
NCT06937567	UCLH801	CAR-T	Advanced solid tumors	No	Phase I	Recruiting	Ongoing – no public clinical results
NCT07152210	CDH17/GUCY2C CAR-T	CAR-T	Colorectal cancer	Yes (GUCY2C)	Phase I	Recruiting	Ongoing – no public clinical results
NCT05411133	Cabotamig (ARB202)	Bispecific antibody	GI malignancies	Yes (CD3)	Phase I	Recruiting	Ongoing – no public clinical results
NCT04137289	BI 905,711	Bispecific antibody	GI cancers	Yes (TRAILR2)	Phase I	Active	Early clinical rationale – AACR abstract: 10.1158/1538-7445.AM2019-2051
NCT05087992	BI 905,711 + chemotherapy	Combination therapy	Colorectal cancer	Yes (TRAILR2)	Phase I	Recruiting	Ongoing – no public clinical results
NCT05948826	TORL-3-600	ADC	Advanced solid tumors	No	Phase I	Recruiting	Ongoing – no public clinical results
NCT06859762	YL217	ADC	Advanced solid tumors	No	Phase I	Recruiting	Ongoing – no public clinical results
NCT07066657	MRG007 (ARR-217)	ADC	Advanced solid tumors	No	Phase I	Recruiting	Ongoing – no public clinical results
NCT07216560	7MW4911	ADC	GI cancers	No	Phase I/II	Recruiting	Ongoing – no public clinical results

*ADC* antibody–drug conjugate, *AACR* American Association for Cancer Research, *ASCO* American Society of Clinical Oncology, *CAR-T* chimeric antigen receptor T-cell therapy, *CDH17* cadherin-17, *CD3* cluster of differentiation 3, *CRC* colorectal cancer, *DOI* digital object identifier, *FIH* first-in-human, *GI* gastrointestinal, *GUCY2C* guanylate cyclase 2 C, *NETs* neuroendocrine tumors, *NCT* National Clinical Trial (ClinicalTrials.gov identifier), *TRAILR2* TNF-related apoptosis-inducing ligand receptor 2

## Discussion & perspectives

Cadherin-17 has emerged as a multifunctional molecule at the interface of adhesion, signaling, tumor differentiation, and therapeutic vulnerability in digestive cancers. Although structurally distinct from classical cadherins due to the absence of canonical catenin-binding domains, CDH17 integrates into major oncogenic signaling networks, particularly Wnt/β-catenin, and intersects with integrin-dependent pathways, stemness programs, and metastatic competence.

A key translational question is whether CDH17 targeting may provide a dual therapeutic benefit, combining selective vectorization of cytotoxic or immune-based agents with direct inhibition of CDH17-driven oncogenic signaling. The study by Bartolomé et al. [121] provided a pivotal demonstration that CDH17 constitutes a functional oncogenic driver in colorectal cancer. This work established CDH17 inhibition itself as a therapeutic strategy, beyond its exploitation as a targeting vector. This functional dimension is particularly relevant in metastatic CRC, where CDH17 has been shown to participate in metastatic outgrowth [56, 67]. Targeting CDH17 may therefore be especially meaningful in the context of colorectal liver metastases, although recent transcriptomic analyses suggest that low-level expression in normal liver parenchyma should be carefully considered [126].

Beyond metastasis, CDH17’s position upstream of Wnt signaling and its intersection with integrin, MYC, and stemness pathways raise the hypothesis that its inhibition could modulate treatment sensitivity. Given the established role of Wnt activation in chemo- and radioresistance, as well as in DNA damage response regulation [127, 128], CDH17 blockade might contribute to sensitization to conventional treatments in Wnt-driven gastrointestinal malignancies. In line with this hypothesis, Bartolomé et al. demonstrated that CDH17 regulates LGR5-dependent Wnt signaling and downstream drug-resistance pathways, including the glutamine transporter SLC38A5 [57]. While the impact of CDH17 inhibition on radiosensitivity remains to be experimentally validated, these data provide a compelling rationale for combining CDH17-targeted strategies with chemotherapy or radiotherapy.

In parallel, emerging data suggest that CDH17 may influence immune regulation. Exosome-derived CDH17 signatures in LUAD and LUSC have been associated with reduced antitumor immune responses [48], and Wnt pathway activation has been linked to immune exclusion in multiple tumor types [129, 130]. Although mechanistic data remain limited, these observations raise the possibility that CDH17 contributes to immune escape mechanisms. This dimension of CDH17 biology remains underexplored and represents a promising area for future investigation.

From a diagnostic perspective, CDH17 appears as a valuable complementary marker in digestive cancers, particularly for metastatic lesions of unknown primary origin. While its performance is largely comparable to CDX2 in gastrointestinal tumors [81–84, 86], its retention in certain CDX2-negative contexts supports its complementary use. However, loss of CDH17 during EMT or dedifferentiation limits its reliability as a standalone biomarker. Importantly, CDH17 expression is not strictly confined to digestive tumors, as detectable levels have been reported in subsets of lung and gynecologic malignancies [34], underscoring the need for careful lineage interpretation.

Interestingly, in parallel with therapeutic strategies, innovative noninvasive diagnostic approaches targeting CDH17 are rapidly emerging. Radiolabeled antibodies and nanobody-based tracers have demonstrated selective tumor detection with high contrast in preclinical models [131–133]. ImmunoPET approaches using 89Zr-DFO-D2101 or 68Ga/18F-labeled nanobodies enabled precise visualization of primary and metastatic lesions, with excellent specificity confirmed by IHC and flow cytometry. These imaging modalities position CDH17 as a promising target for molecular staging and occult metastasis detection.

Importantly, the convergence of therapeutic and imaging applications highlights the theranostic potential of CDH17. Radiolabeled nanobodies, CDH17-directed CAR T and CAR NK cells [112, 134], bispecific ADCs such as BI 905,711, immunotoxins, and nanobody-functionalized extracellular vesicles collectively demonstrate that CDH17 can serve both as a functional therapeutic target and as a selective delivery platform. The restricted physiological expression of CDH17 to basolateral intestinal epithelium, combined with its aberrant overexpression and mislocalization in tumors, supports a favorable therapeutic index. Nevertheless, heterogeneous expression and loss in EMT contexts necessitate pre-treatment biomarker evaluation, such as CDH17 IHC on tumor biopsies.

Finally, CDH17’s therapeutic target potential is reflected in ongoing drug development efforts. Over 30 programs are currently focused on CDH17, primarily exploring ADCs, CAR T cells, and T-cell engagers. Ten of these agents have advanced into clinical trials (Table 4). Notable examples include Lepu/Arrivent’s MRG007 [135], Sotio/Biocytogen’s SOT109 [136], and MediLink’s YL217 [137], all presented at the AACR 2025 conference. Several other candidates are progressing toward investigational new drug (IND) filings, such as Huadong Medicine’s HDM2017 [138] and VelaVigo’s VBC108 [139], a bispecific ADC targeting both CDH17 and Claudin18.2.

## Conclusion

**Fig. 5:**
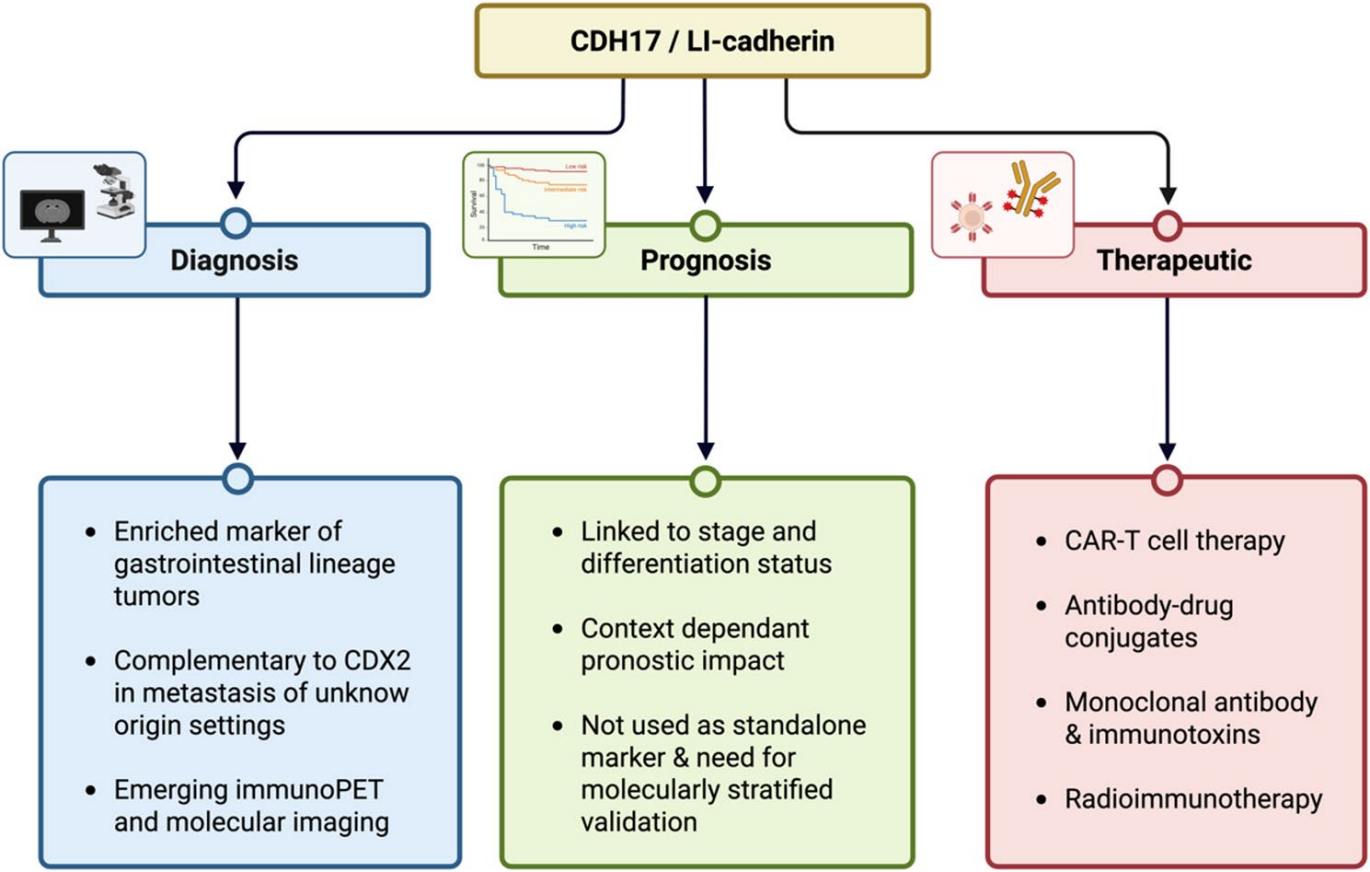
Translational roles of CDH17/LI-cadherin in digestive oncology. Schematic representation of the multifaceted roles of CDH17 in digestive tumor management. CDH17 is explored for its high diagnostic specificity, potential prognostic relevance, and promising applications in targeted therapies strategies. Figure created with BioRender.com

Altogether, CDH17 emerges as a highly versatile molecule at the crossroads of differentiation, tumor plasticity, immune modulation, and therapeutic vulnerability. Its restricted physiological expression and tumor-specific deregulation make it particularly attractive for next-generation diagnostic, imaging, and targeted therapeutic strategies (Fig. 5). Future studies should prioritize biomarker-driven patient stratification, longitudinal monitoring of CDH17 dynamics, and the integration of CDH17-targeted approaches into combinatorial treatment regimens. Such efforts will be essential to fully exploit CDH17 as a lever for precision oncology in gastrointestinal and potentially non-digestive malignancies.

## Methods

To identify relevant articles for this literature review, a systematic search was conducted using the keywords “LI-cadherin”, “liver-intestine cadherin”, “CDH17”, and “cadherin 17”, to ensure comprehensive coverage of the topic. The search was primarily carried out through the PubMed database, with additional searches performed on Google to capture other relevant scientific sources, until January 2026 included. Articles were screened based on their relevance to the biological functions, clinical significance, and potential therapeutic implications of CDH17.

## Data Availability

The data that support the findings of this study are available on request from the corresponding author, A.S.

## Abbreviations

ADC: Antibody–drug conjugate
AFP: Alpha–fetoprotein
AKT: Protein kinase B
ASCO: American Society of Clinical Oncology
BsADC: Bispecific antibody–drug conjugate
BilIN: Biliary intraepithelial neoplasia
CAR: NK–Chimeric antigen receptor natural killer cells
CAR: T–Chimeric antigen receptor T cells
CCND1: Cyclin D1
CDH17: Cadherin 17
CDX: Cell line–derived xenograft
ChIP: seq–Chromatin immunoprecipitation sequencing
CRC: Colorectal cancer
CT: Cytoplasmic tail
CTC: Circulating tumor cells
DFS: Disease–free survival
DOI: Digital Object Identifier
DSB: Double–strand break
EC: Extracellular domain
ELISA: Enzyme–linked immunosorbent assay
EMT: Epithelial–mesenchymal transition
ERK: Extracellular signal–regulated kinase
EVs: Extracellular vesicles
FAK: Focal adhesion kinase
FIH: First–in–human
GC: Gastric cancer
GI: Gastrointestinal
GPX4: Glutathione peroxidase 4
GSK3β: Glycogen synthase kinase 3 beta
GUCY2C: Guanylate cyclase 2 C
HCC: Hepatocellular carcinoma
HR: Homologous recombination
ICG: Indocyanine green
iCCA: Intrahepatic cholangiocarcinoma
IHC: Immunohistochemistry
JNK: c–Jun N–terminal kinase
LNMM: Lymph node micrometastasis
LNM: Lymph node metastasis
LUAD: Lung adenocarcinoma
MAPK: Mitogen–activated protein kinase
mAb: Monoclonal antibody
MMAE: Monomethyl auristatin E
MSI: Microsatellite instability
MYC: MYC proto–oncogene
NCT: National Clinical Trial (ClinicalTrials.gov identifier)
NF: κB–Nuclear factor kappa–light–chain–enhancer of activated B cells
NHEJ: Non–homologous end joining
NHP: Non–human primates
NIR: Near–infrared
NET: Neuroendocrine tumor
OS: Overall survival
PanNET: Pancreatic neuroendocrine tumor
PDAC: Pancreatic ductal adenocarcinoma
PDX: Patient–derived xenograft
PI3K: Phosphoinositide 3–kinase
PLC: Primary liver cancer
RFS: Recurrence–free survival
RNA: seq–RNA sequencing
RT: qPCR–Reverse transcription quantitative polymerase chain reaction
RRx: 001–Dinitroazetidine derivative radiosensitizer
SPECT/CT: Single–photon emission computed tomography / Computed tomography
TAZ: Transcriptional coactivator with PDZ–binding motif
TRAILR2: TNF–related apoptosis–inducing ligand receptor 2
WDNET: Well–differentiated neuroendocrine tumor
Wnt: Wingless–related integration site signaling pathway
YAP: Yes–associated protein
